# Preoperatively Grading Rectal Cancer with the Combination of Intravoxel Incoherent Motions Imaging and Diffusion Kurtosis Imaging

**DOI:** 10.1155/2020/2164509

**Published:** 2020-10-12

**Authors:** Zhijun Geng, Yunfei Zhang, Shaohan Yin, Shanshan Lian, Haoqiang He, Hui Li, Chuanmiao Xie, Yongming Dai

**Affiliations:** ^1^Department of Radiology, Sun Yat-sen University Cancer Center, State Key Laboratory of Oncology in Southern China, No. 651 Dongfeng East Road, Guangzhou 510060, China; ^2^Central Research Institute, United Imaging Healthcare, Shanghai 201807, China

## Abstract

**Purpose:**

To combine Intravoxel Incoherent Motions (IVIM) imaging and diffusion kurtosis imaging (DKI) which can aid in the quantification of different biological inspirations including cellularity, vascularity, and microstructural heterogeneity to preoperatively grade rectal cancer.

**Methods:**

A total of 58 rectal patients were included into this prospective study. MRI was performed with a 3T scanner. Different combinations of IVIM-derived and DKI-derived parameters were performed to grade rectal cancer. Pearson correlation coefficients were applied to evaluate the correlations. Binary logistic regression models were established via integrating different DWI parameters for screening the most sensitive parameter. Receiver operating characteristic analysis was performed for evaluating the diagnostic performance.

**Results:**

For individual DWI-derived parameters, all parameters except the pseudodiffusion coefficient displayed the capability of grading rectal cancer (*p* < 0.05). The better discrimination between high- and low-grade rectal cancer was achieved with the combination of different DWI-derived parameters. Similarly, ROC analysis suggested the combination of *D* (true diffusion coefficient), *f* (perfusion fraction), and *K*_app_ (apparent kurtosis coefficient) yielded the best diagnostic performance (AUC = 0.953, *p* < 0.001). According to the result of binary logistic analysis, cellularity-related *D* was the most sensitive predictor (odds ratio: 9.350 ± 2.239) for grading rectal cancer.

**Conclusion:**

The combination of IVIM and DKI holds great potential in accurately grading rectal cancer as IVIM and DKI can provide the quantification of different biological inspirations including cellularity, vascularity, and microstructural heterogeneity.

## 1. Introduction

It has been reported that there were around 0.7 million new cases of rectal cancer, accounting for approximately 40% of 1.8 million new colorectal cancer cases in 2018. Moreover, rectal cancer has posed a huge threat to human health because of high mortality rates (∼0.2 million deaths in 2018) [1]. Several factors including mesorectal fascia, stage, histopathologic grade, and vascular invasion are tightly correlated with the prognosis of rectal cancer [2, 3]. Currently, the widespread therapeutic option for rectal cancer, neoadjuvant chemoradiotherapy, may inevitably lead to some serious side effects, especially when accurate evaluation of histopathological grade is not available [2, 4]. Therefore, noninvasive and accurate evaluation of the histopathologic grade of rectal cancer is of great clinical importance for directing subsequent clinical management.

Due to the clinical significance of providing different biological inspirations, Diffusion-Weighted Magnetic Resonance Imaging (DW-MRI) has shown tremendous clinical potential. During the past few decades, much effort has been made to propose novel DWI models such as intravoxel incoherent motion (IVIM) [5], diffusion kurtosis imaging (DKI) [6], fractional order calculus (FROC) [7], and restriction spectrum imaging (RSI) [8] for characterizing tumor from different perspectives via different DWI-derived biological inspirations. Interestingly, although there are numerous DWI models, DWI-derived biological inspirations mainly contain cellularity, vascularity, and microstructural heterogeneity [9]. For instance, the apparent diffusion coefficient (ADC) sourced from conventional DWI provides the biological inspiration of cellular density which will increase with the progression of tumors. Differently, the DKI-derived apparent kurtosis coefficient (*K*_app_) indirectly represents the microstructural complexity in comparison to IVIM-derived *f* (perfusion fraction) that can quantify the vascularity [5]. Hence, combining these DWI-derived biological inspirations will pave the way for more comprehensively characterizing tumors from different perspectives. Numerous researchers have simultaneously combined multiple DWI models for achieving clinical objectives such as tumor diagnosis, staging, and grading [10–12]. However, innumerable attention has been paid to comparing the clinical effectiveness of different DWI-derived parameters, which aimed to explore the best DWI-derived parameter or DWI model for specific clinic purpose. The combination of different DWI models was hardly performed based on the DWI-derived biological inspiration. For example, Bai et al. compared the diagnostic value of different parameters calculated from monoexponential, biexponential, and stretched exponential DWI and DKI for grading glioma [13]. Li performed a comparative study of Gaussian and non-Gaussian DWI models for differential diagnosis of prostate cancer [14]. Moreover, excessive DWI models may weaken the clinical potential because of the long scanning time, low patient compliance, and difficult manipulation and postprocessing. Based on the aforementioned points, we hypothesized that (1) the combination of IVIM and DKI was sufficient to provide three main DWI-derived biological inspirations including cellularity, vascularity, and microstructural complexity. (2) Integrating these DWI-derived biological inspirations together will benefit the accurate grading of rectal cancer through more comprehensive tumor characterization. Thus, this research aimed to combine IVIM and DKI to grade rectal cancer via integrating different DWI-derived biological inspirations. Moreover, the correlations among the DWI-derived cellularity, vascularity, and microstructural complexity were also investigated. As far as we are aware, hardly has the integration of different DWI-derived biological inspirations been performed to grade rectal cancer.

## 2. Methods

### 2.1. Patients

The approval from a local institute review board was obtained for this prospective research. Written informed consent was obtained from all patients. A total number of 69 patients were recruited into this prospective research between December 2018 and August 2019. The inclusion criteria and exclusion criteria were established according to a previous research [2] and are listed as the follows:

Inclusion criteria:

1. Endoscopic biopsy-proven rectal cancer
2. More than one week of interval between biopsy and MRI

Exclusion criteria:

1. Poor quality of DKI or IVIM images caused by artifacts
2. Patients who underwent surgery before the MRI examination
3. Time interval between MR examination and surgery of more than 2 weeks
4. Preoperative neoadjuvant chemoradiotherapy
5. Inaccessible clinical pathology results of histopathologic grade

### 2.2. MRI Protocols

All MRI measurements were performed with a 3T whole-body scanner (uMR 780, United Imaging Healthcare Co., Ltd.) with a twelve-channel coil. The MRI protocol mainly included a T2-weighted Fast Spin Echo sequence termed as FSE T2WI (echo time/repetition time, 103.1/4244.0 ms/ms; flip angle, 110°; FOV, 280 × 360 mm^2^; matrix, 336 × 432; slice thickness, 6 mm; intersection gap, 1.2 mm; and number of slices, 25), dynamic three-dimensional T1 weighted gradient echo (GRE) sequence (echo time/repetition time, 1.45/3.30 ms/ms; flip angle, 10°; FOV 280 × 500 mm^2^; and matrix, 336 × 480.), and oblique axial Single Shot- Echo Planar Imaging sequence termed as SS-EPI (echo time/repetition time, 86.2/4600.0 ms/ms; flip angle, 90°; FOV 180 × 240 mm^2^; matrix, 168 × 224; slice thickness, 4 mm; intersection gap, 1 mm; number of slices, 20; *b* values: 0, 10, 20, 30, 50, 80, 100, 150, 200, 400, 600, 800, 1500, and 2000 s/mm^2^; and scanning time: 4.7 min). It should be noted that both DKI and IVIM were based on the abovementioned SS-EPI sequence with different selections of *b* values for subsequent postprocessing.

### 2.3. Image Analysis

All the original data were processed with one in-house prototype software developed by MATLAB (Mathworks, Natick, Mass).

#### 2.3.1. IVIM

The quantitative pixelwise parameters derived from IVIM were obtained through the previously-reported fitting model [5]:

(1) $$\begin{array}{c} \frac{S_{b}}{S_{0}}=\left(1-f\right)\exp\left(-b\;D\right)+f\;\exp\left(-bD^{*}\right), \end{array}$$

where *S*_0_ and *S*_*b*_ are, respectively, the signal intensity when a *b* value of 0 s/mm^2^ and other *b* values are applied. *f* is the perfusion volume fraction, *D* (unit: ×10^−9^ m^2^/s) is the true diffusion coefficient representing pure diffusion, and *D*^*∗*^ (unit: ×10^−9^ m^2^/s) is the pseudodiffusion coefficient representing perfusion related diffusion (incoherent microcirculation within the voxel). Moreover, the fitting of IVIM was based on the images of *b* values of 0, 10, 20, 30, 50, 80, 100, 150, 200, 400, 600, and 800 s/mm^2^.

#### 2.3.2. DKI

The quantitative pixelwise parameters derived from the DKI were obtained through the previously-reported fitting model [6]:

(2) $$\begin{array}{c} \ln\left(S_{b}\right)=\ln\left(S_{0}\right)-b\cdot D_{\text{app}}+\frac{1}{6}\cdot b^{2}D_{\text{app}}^{2}K_{\text{app}}, \end{array}$$

where *S*_*b*_ and *S*_0_ are identical to those in IVIM. *D*_app_ (unit: ×10^−9^ m^2^/s) and *K*_app_ (unitless) are, respectively, the apparent diffusion coefficient and apparent kurtosis coefficient representing the degree to which molecular motion deviated from the Gaussian diffusion. Additionally, the fitting of DKI was based on the images of *b* values of 0, 800, 1500, and 2000 s/mm^2^.

Two radiologists (ZJ.G and CM.X), with 8 and 25 years' experience of gastrointestinal imaging were asked to draw the Volumes of Interest (VOIs) along the tumor border, which meant that all DWI-derived parameters were measured based on the whole-lesion method, and the entire tumor was maximally included into the VOI. The definitions of each VOI were based on the consensus of the abovementioned two radiologists. Before drawing VOIs, radiologists were blinded to the results of histopathological examination. For each slice within tumor, freehand regions were drawn along the border of the low signal of the tumor on the *D* map with T2WI images as the references. Necrosis, cyst, and haemorrhage were carefully excluded. In this way, the whole tumor was incorporated into the VOI. Then, the outlined regions were automatically copied to other parametric maps including *f* map, *D*^*∗*^ map for IVIM, and *D*_app_ and *K*_app_ map for DKI. Finally, the pixel-based average values for each parameter were acquired by means of the whole tumor averaging approach reported before [15].

### 2.4. Histopathological Evaluation

All pathological examinations were concluded by an experienced pathologist with more than 5 years' experience. Surgical specimens of rectal cancer were routinely prepared into 5 *μ*m slices and, then, stained with hematoxylin-eosin (H&E). Histological grading was performed according to the WHO criteria [16]. Rectal cancer patients were classified as grade 1 (G1), grade 2 (G2), or grade 3 (G3) when gland-like structures of the tumor occupied greater than 95%, greater than 50% but less than or equal to 95%, or less than or equal to 50% of the volume, respectively.

### 2.5. Statistical Analysis

Firstly, the Kolmogorov–Smirnov test was performed for analyzing normality. According to the result of the Kolmogorov–Smirnov test, independent Student's *t*-test was applied to see whether there existed significant differences between different groups (low grade (G1-2) and high grade (G3)). Moreover, one-way ANOVA and the Tukey post hoc test were performed for multiple comparison of quantitative diffusion parameters among the groups of G1, G2, and G3 rectal cancer. The Pearson correlation test was performed to assess the correlation coefficients abbreviated as *r* between the parameters. Binary logistic regression analysis was performed to establish the diagnostic model with the combination of different parameters including *D* (cellularity), *f* (vascularity), and *K*_app_ (microstructural complexity) of IVIM and DKI which showed significant differences between low-grade and high-grade rectal cancer groups for subsequent ROC analysis. *D*^*∗*^ was excluded because it did not display a significant difference between the low-grade rectal cancer and high-grade rectal cancer. Although *D*_app_ also displayed a significant difference between the low-grade and high-grade rectal cancer, it was excluded because of the following issues: (1) *D*_app_ possesses the same biological insight of cellularity as *D*. (2) Eliminating the influence of perfusion, *D* is better at characterizing the true diffusion restriction resulted from cellularity [17]. As a result, the diagnostic model-based combinations of *D* and *f* (cellularity and vascularity), *D* and *K*_app_ (cellularity and microstructural complexity), and *f* and *K*_app_ (vascularity and microstructural complexity), as well as *D* and *f* and *K*_app_ (cellularity and vascularity and microstructural complexity), were established via logistic regression. Besides introducing three variables (*D*, *f*, and *K*_app_), binary logistic regression analysis was also performed to evaluate which DWI-derived biological insight among cellularity, vascularity, and microstructure was the most sensitive for predicting high-grade rectal cancer by comparing the standardized regression coefficients (*β*) and the odds ratio (OR) of different parameters. The OR was calculated according to the following formula: OR=exp(|*β*|). In order to obtain the standardized regression coefficients of binary logistic regression analysis, *D*, *f*, and *K*_app_ were, firstly, standardized as the *Z* score to eliminate the effect of dimension and quantity of data. It is worthwhile to be noted that, for obtaining more intuitive comparison, the odds ratio of *f* and *D* was defined as the ratio of the positive (high-grade rectal cancer in this research) probability after the variable decreased by one standard unit to the probability before the change, which was different from the standard definition. The definition of the odds ratio of *K*_app_ was the same as the standard definition. The ROC (receiver operating characteristic curve) analysis was performed to evaluate the diagnostic performance of parameters showing significant differences between low-grade and high-grade rectal cancer groups together with their combinations by comparing the AUCs (area under curve). All parameters in this research were statistically analyzed with statistical tools including SPSS software (PASW Statistics 25.0 SPSS Inc., Chicago, IL, USA), Medcalc (MedCalc 9.0.2, Mariakerke, Belgium), *R* version 3.6.1 (R Core Development Team), and RStudio (RStudio Inc, Boston, MA, USA). It was regarded as having a statistical significance when the *p* value was less than 0.05.

## 3. Results

Between December 2018 and August 2019, a total of 69 patients were initially recruited into this prospective study. Three patients were excluded because of poor quality of MR images. Two patients were excluded because they underwent surgery before the MR examination. In addition, two patients were excluded because of the preoperative neoadjuvant chemoradiotherapy. Four patients were excluded as the pathological results of histopathologic grade were inaccessible. Ultimately, 58 patients (59.3 ± 10.2 years, male:33, female: 25) were included for subsequent analysis. Of the 58 patients, 11 were classified as WHO G1, 29 were classified as WHO G2, and 18 patients were classified as WHO G3. The clinical data of 58 patients are presented in Table 1.

### 3.1. Correlations between the DWI-Derived Parameters and Histopathologic Grade

**Figure 1:**
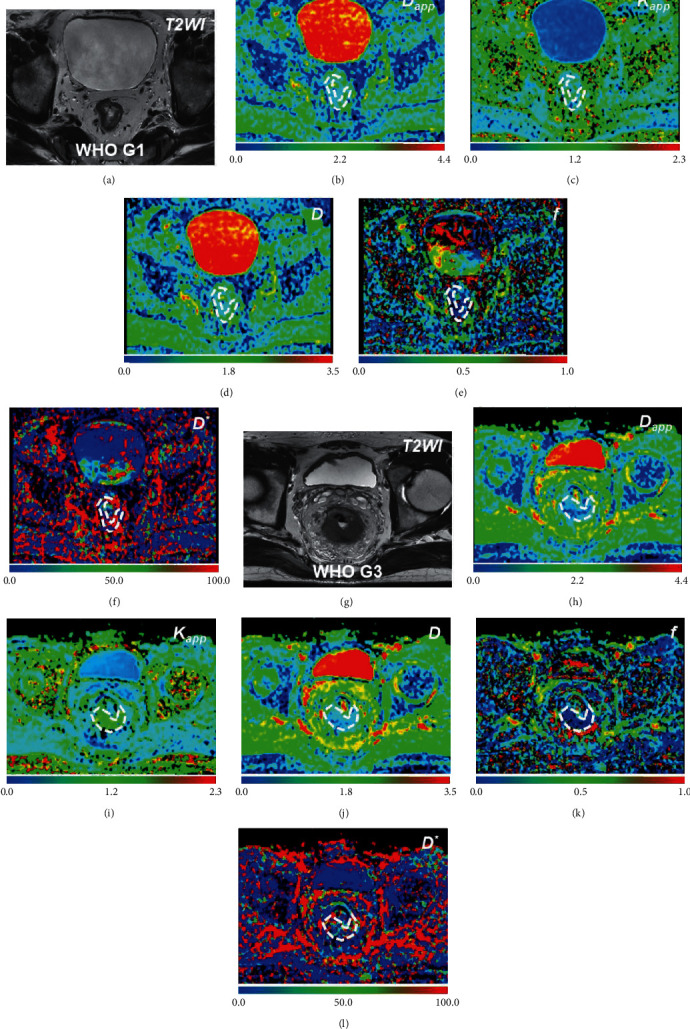
MR images of patients with surgically proven rectal cancer of WHO G1 (a–f) and WHO G3 (g–l). A patient of WHO G1 rectal cancer: (a) T2-weighted image, (b) *D*_app_ image, (c) *K*_app_ image, (d) *D* image, (e) *f* image, and (f) *D*^*∗*^ image. A patient of WHO G3 rectal cancer: (g) T2-weighted image, (h) *D*_app_ image, (i) *K*_app_ image, (j) *D* image, (k) *f* image, and (l) *D*^*∗*^ image. *Note.* The unit of *D D*^*∗*^ and *D*_app_: ×10^−9^ m^2^/s; *K*_app_ and *f* are unitless.

Representative MR images of a patient with WHO G1 rectal cancer and a patient with WHO G3 rectal cancer are displayed in Figure 1.

#### 3.1.1. Individual DWI-Derived Parameters

**Figure 2:**
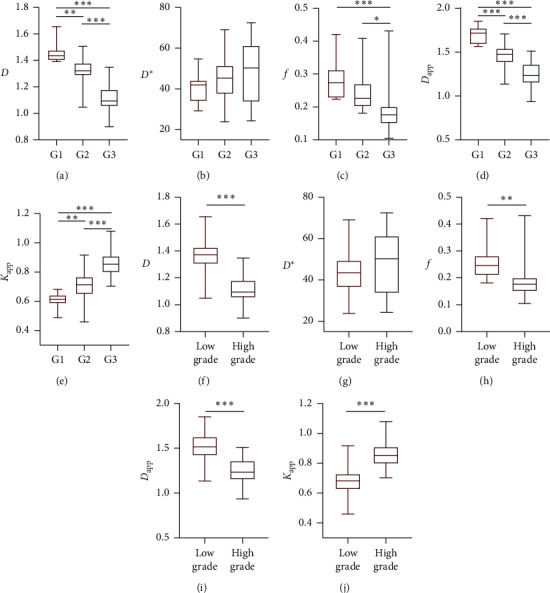
Grading rectal cancer with individual DWI-derived inspiration-based parameters. (a) *D* value (unit: ×10^−9^ m^2^/s), (b) *D*^*∗*^ (unit: ×10^−9^ m^2^/s), (c) *f* value, (d) *D*_app_ value (unit: ×10^−9^ m^2^/s), (e) *K*_app_ value of WHO G1, WHO G2, and WHO G3 rectal cancer. (f) *D* value (unit: ×10^−9^ m^2^/s), (g) *D*^*∗*^ value (unit: ×10^−9^ m^2^/s), (h) *f* value, (i) *D*_app_ value (unit: ×10^−9^ m^2^/s), (j) *K*_app_ value of low-grade (WHO G1-2) and high-grade (WHO G3) rectal cancer. *Note.*^*∗*^*p* < 0.05, ^*∗∗*^*p* < 0.01, and ^*∗∗∗*^*p* < 0.001. *f* and *K*_app_ is unitless.

The results of directly grading rectal cancer patients by individual DWI parameters including *D* and *D*_app_ (cellularity), *D*^*∗*^ and *f* (vascularity), and *K*_app_ (microstructural complexity) are presented in Figure 2 via boxplots. All quantitative parameters except *D*^*∗*^ showed the capability of discriminating between rectal patients of different grades with significant difference (*p* < 0.05). As the histopathologic grade increased, *D* (G1: 1.465 ± 0.081, G2: 1.323 ± 0.105, G3: 1.105 ± 0.103, low grade (G1 and G2): 1.362 ± 0.117 and high grade (G3): 1.105 ± 0.103. Unit: ×10^−9^ m^2^/s), *D*_app_ (G1: 1.699 ± 0.099, G2: 1.460 ± 0.127, G3: 1.250 ± 0.144 low grade: 1.526 ± 0.161 and high grade: 1.250 ± 0.144. Unit: ×10^−9^ m^2^/s), and *f* (G1: 0.292 ± 0.067, G2: 0.244 ± 0.052, G3: 0.192 ± 0.072, low grade: 0.257 ± 0.059 and high grade: 0.192 ± 0.072) decreased, but *K*_app_ (G1: 0.604 ± 0.058, G2: 0.715 ± 0.091, G3: 0.862 ± 0.099, low grade: 0.684 ± 0.096 and high grade: 0.862 ± 0.099) increased. Detailed comparisons and significance levels are presented in Table 2. Interestingly, compared to others, *f* showed a weaker capability of grading rectal cancer as it not only displayed no significant difference between G1 and G2 (*p*_G1 vs G2_ = 0.079) but also smaller difference (*p*_G2 vs G3_ = 0.018, *p*_low−vs. high grade_ = 0.002) between the subgroups compared to other parameters.

#### 3.1.2. Combinations of Different DWI-Derived Parameters

**Figure 3:**
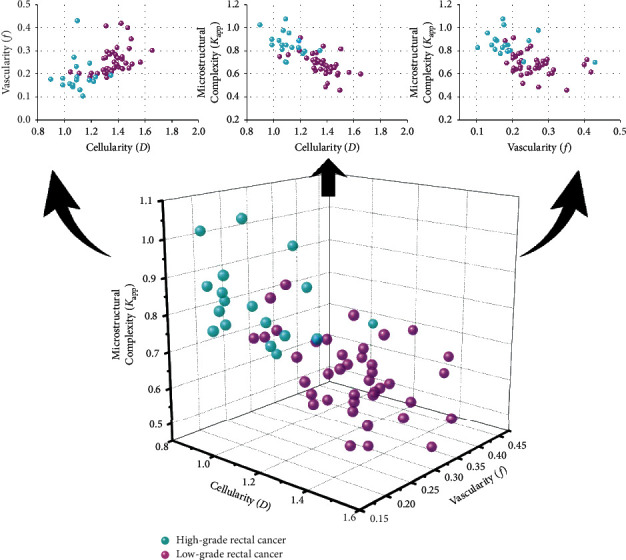
Discriminating the high-grade rectal cancer from low-grade rectal cancer patients in 2D data spaces constructed by the combination of different DWI-derived biological inspirations (first row: *D* (cellularity) and *f* (vascularity), *D* (cellularity) and *K*_app_ (microstructural complexity) and f (vascularity) and *K*_app_ (microstructural complexity) and 3D space constructed by all DWI-derived inspirations (second row: *D* (cellularity) and *f* (vascularity) and *K*_app_ (microstructural complexity)). *Note*. Each point in the figure represents a patient with rectal cancer.

The first row of Figure 3 shows that 2D data spaces were constructed by different combinations of DWI-derived parameters, i.e., *D* and *f*, *D* and *K*_app_, *f* and *K*_app_, and *D* and *f* and *K*_app_. The results in the first row of Figure 3 demonstrate that a clearer distinction between high- and low-grade rectal cancer patients was achieved via introducing second DWI-derived biological insight with regard to a single DWI-derived biological insight. Obviously, the high-grade patients were much better separated from low-grade patients in the 3D data space when cellularity (*D*), vascularity (*f*), and microstructural complexity (*K*_app_) were simultaneously integrated with small data overlap.

### 3.2. Screening the Most Sensitive DWI-Derived Biological Insight for Grading Rectal Cancer

**Figure 4:**
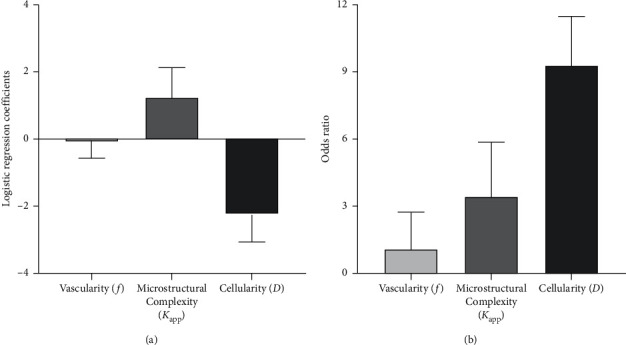
Screening of the most sensitive DWI-derived biological insight from cellularity, vascularity, and microstructural complexity. (a) The standardized logistic regression coefficient of vascularity *f*, microstructural complexity (*K*_app_), and cellularity *D*. (b) Odds ratios of vascularity *f*, microstructural complexity (*K*_app_), and cellularity *D*. Notes: (1) the odds ratio of microstructural complexity (*K*_app_) is 3.440, indicating when *K*_app_ increases by one unit, the probability of suffering from high-grade rectal cancer is as 3.440 times high as before. (2) The odds ratio of cellularity *D* was 9.350, indicating that when *D* decreased by one standard unit, the probability of suffering from high-grade rectal cancer was as 9.350 times high as before.

A binary logistic regression model was established by introducing three variables: *D*, *f*, and *K*_app_. The standardized regression coefficients (*β*) of different DWI-derived biological inspirations are presented in Figure 4(a) (*β*_cellularity_ = −2.235 ± 0.806, *β*_vascularity_ = −0.081 ± 0.527, and *β*_microstructural complexity_ = 1.238 ± 0.905). The odds ratios (OR) of different DWI-derived biological insight are presented in Figure 4(b) (OR_cellularity_ = 9.350 ± 2.239, OR_vascularity_ = 1.084 ± 1.694, and OR_microstructural complexity_ = 3.440 ± 2.472). The OR of microstructural complexity was 3.440, indicating that when *K*_app_ increased by one standard unit, the probability of high-grade rectal cancer was 3.440 times as high as before. The odds ratio of cellularity was 9.350, indicating when *D* decreased by one standard unit, the probability of high-grade rectal cancer was 9.350 times as high as before. It should be noted that the definition of OR_cellularity_ and OR_vascularity_ was different from the standard definition of OR. Detailed definitions can be found in the Section 2.

### 3.3. Diagnostic Performance Evaluation

**Figure 5:**
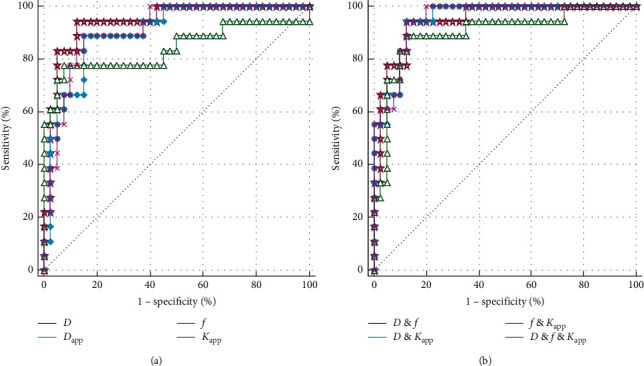
Diagnostic performance evaluation. (a) Diagnosis of high-grade rectal cancer with single DWI-derived biological insight-based parameters including cellularity (*D* and *D*_app_) and vascularity *f* together with microstructural complexity (*K*_app_). (b) Diagnosis of high-grade rectal cancer with the combinations of different DWI inspirations including cellularity and vascularity (*D* and *f*, cellularity and microstructural complexity (*D* and *K*_app_), vascularity and microstructural complexity (*f* and *K*_app_), and cellularity and vascularity and microstructural complexity (*D* and *f* and *K*_app_).

Figure 5 demonstrates the diagnostic performance of different parameters and their combinations. The area under curve (AUC) and other indexes are listed in Table 3. Briefly, the following AUCs are listed in order from large to small: AUC _*D* (cellularity) & *f* (vascularity) & *K*app (microstructural complexity)_ = 0.953, AUC _*D* (cellularity) &__*K*app (microstructural complexity)_ = 0.951, AUC _*D* (cellularity) & *f* (vascularity)_ = 0.946, AUC _*D* (cellularity)_ = 0.912, AUC _*K*app (microstructural complexity)_ = 0.910, AUC _*f* (vascularity) & *K*app (microstructural complexity)_ = 0.901, AUC_*D*app (cellularity)_ = 0.901, and AUC _*f* (vascularity)_ = 0.843. All the abovementioned AUCs were significantly different from the AUC of 0.500 (*p* < 0.001).

### 3.4. Exploring the Correlation between Different DWI-Derived Biological Inspirations

**Figure 6:**
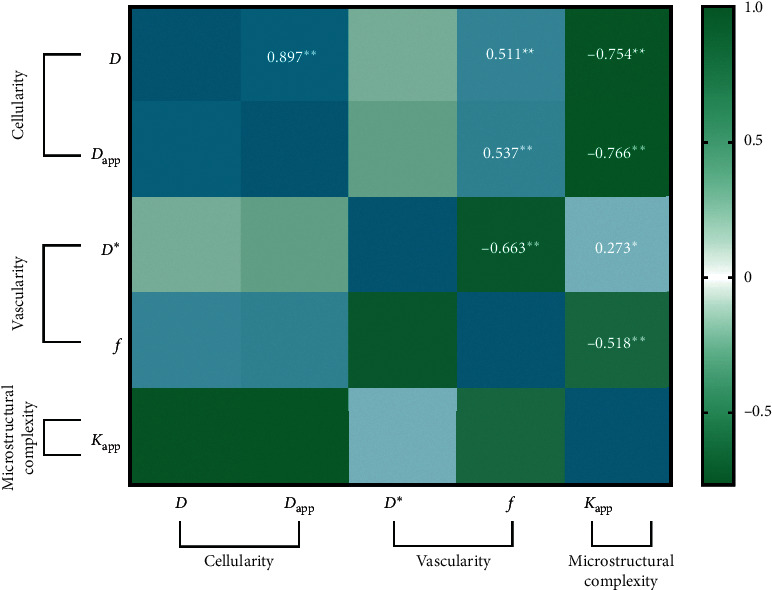
Heat map depicting the Pearson correlation between cellularity characterized by *D* and *D*_app_ and vascularity characterized by *D*^*∗*^ and f, as well as microstructural complexity characterized by *K*_app_. *Notes*. The numbers represent the Pearson coefficient. ^*∗*^*p* < 0.05, ^*∗∗*^*p* < 0.01, and ^*∗∗∗*^*p* < 0.001.

The binary correlations between different DWI-derived biological inspirations were evaluated via Pearson correlation coefficients abbreviated as *r*, which are presented in Figure 6. *D* or *D*_app_ were significantly and positively correlated with *f* (*r*_*D* & *f* = 0.511,_*p* < 0.01_;_*r*_*D*app & *f* = 0.537,_*p* < 0.01) but negatively correlated with *K*_app_ (*r*_*D* & *K*app = −0.754,_*p* < 0.01_;_ r_*D*app & *K*app = −0.766,_*p* < 0.01). Besides, *f* was negatively correlated with *K*_app_ (*r*_*f* & *K*app = −0.518,_*p* < 0.01). Interestingly, only f and Kapp showed significant correlations with D^*∗*^ (*r*_*D*_^*∗*^_& *f* = −0.663,_*p* < 0.01_;_*r*_*D*_^*∗*^_& *K*app = 0.273_, *p* < 0.05).

## 4. Discussion

In comparison to plenty of previous studies that simultaneously combined many DWI models but focused more on comparing the clinical effectiveness of different parameters that were calculated from various DWI models such as ADC, *f*, and *D* for tumor grading, staging, and so on, important highlights of this research are the followings: (1) combining DWI models according to their biological inspirations will benefit the accurate grading of rectal cancer via more comprehensive tumor characterization. (2) The combination of DKI and IVIM was enough to provide three main DWI-derived biological inspirations, which can avoid the disadvantages caused by excessive DWI models such as long scan time, difficult postprocessing and manipulation, and low patient compliance. (3) The results of the present research suggest that all three DWI-derived biological inspirations are tightly correlated with each other, which further proved the necessity of combining these DWI-derived inspirations together. (4) The results indicate that DWI-derived cellularity was the most sensitive for grading rectal cancer followed by microstructural complexity and vascularity.

For individual parameters, individual DWI-derived biological insight-based parameters except *D*^*∗*^ all had the capability of distinguishing the high-grade from low-grade rectal cancer. The biological basis is as follows: (1) The rapid proliferation of cancer cells leads to an increase in nuclear-to-cytoplasmic ratio and to a decrease in extracellular space, which ultimately results in an increase in the degree of diffusion restriction reflected by a decrease of diffusion coefficient (*D*_app_ and *D*) [18]. (2) Differently, as the histopathologic grade increases, the extent to which the diffuse water molecules deviate from the Gaussian distribution increases, which can be quantified by the increase in the kurtosis coefficient (*K*_app_) [19]. (3) *f* decreased as histopathological grade increased was because of the following reasons: the vascular systems will be severely destroyed together with the macromanifestations such as intratumoral bleeding as tumor proliferates, which resulted in the decrease of *f* representing the perfusion fraction [20]. Based on the aforementioned points, (1) cellularity-, vascularity-, and microstructural complexity-related parameters had the capability for grading rectal cancer. (2) There were significant negative correlations between cellularity-related parameters (*D* and *D*_app_) and microstructural complexity-related parameter (*K*_app_), as well as vascularity-related parameters (*f*) and microstructural complexity-related parameter (*K*_app_). Moreover, cellularity-related parameters (*D* and *D*_app_) significantly and positively correlated with the vascularity-related parameter (*f*). Interestingly, IVIM-derived *D*^*∗*^ displayed a negative correlation with *f* but a positive correlation with *K*_app_, which can be explained by the following points: (1) according to IVIM theory, *D*^*∗*^ is influenced by the several factors that can be expressed as the following equation: *D*^*∗*^=(*l* × *v*)/6, where  *l* represents the capillary length and *v* represents the average velocity of blood in the capillary. (2) As mentioned above, the poor structure of lumenized vessels dominates in high-grade rectal cancer. However, in order to meet the rapidly growing need of oxygen and nutrients for the tumor cells, the average velocity of blood in the capillary will increase compensatively. Therefore, *D*^*∗*^ was significantly positively correlated with *K*_app_ but negatively with *f*. Correlations among DWI-derived cellularity, vascularity, and microstructural complexity further proved the necessity of integrating these inspirations together to achieve a more comprehensive tumor characterization. In addition, the vascularity-related *f* showed the weaker diagnostic power compared to cellularity- and microstructural complexity-related parameters. These results can be explained as follows: (1) *D*^*∗*^ is very vulnerable to the effects of a low signal to noise ratio. (2) The vascularity variation is not as sensitive as cellularity and microstructural complexity during carcinogenesis. (3) Several studies proposed that *f* is not accurate for diagnosing tumor. [17] When different DWI-derived parameters were combined, a better separation of high-grade from low-grade rectal cancer was achieved with less overlap between groups. The causes for abovementioned results were speculated as that more DWI parameters meant a more comprehensive characterization of the tumor, which ultimately led to a better separation in 3D data space via combining different DWI-derived biological inspirations. The ROC analysis results indicated that, among all the parameters and their combinations, the best diagnostic (AUC = 0.953) performance was achieved when all three DWI-derived biological inspirations were simultaneously integrated. Moreover, in general, the diagnostic performance of the combination of two DWI-derived inspiration-based parameters was better than that of single DWI-derived insight-based parameter. As mentioned above, the more the DWI-derived inspirations, the more comprehensive the characterization of rectal cancer, which ultimately resulted in the better diagnostic performance for grading rectal cancer.

The binary logistic regression analysis results demonstrated that cellularity quantified by IVIM-derived *D* was the most sensitive for predicting high-grade rectal cancer, followed by microstructural complexity quantified by DKI-derived *K*_app_ and vascularity quantified by IVIM-derived *f*. The abovementioned results are consistent with the ROC analysis results. The reasons may be the following: (1) Among cellularity, vascularity, and microstructural complexity, the cellularity variation is the most pronounced. Similar to the results of our study, Fujima demonstrated that *D* (25^th^ percentile) served as the most powerful indicator for predicting the treatment outcome of nasal or sinonasal squamous cell carcinoma patients. [21] It was reported by Shirato et al. that the *D* value obtained from IVIM showed a higher value in discriminating distant metastasis compared to the parameters derived from DKI [22]. (2) Some previous studies proposed that *f*, representing the vascularity, is not accurate enough, always leading to mixed results [17, 20, 23]. Interestingly, other researchers have drawn different conclusions. Different from our study, Wang et al. concluded that *K*_app_ is the most valuable diagnostic marker for grading glioma in comparison to the other parameters [13]. Similarly, Zhu's research concluded that *K*_app_ is much more effective in grading and evaluating the proliferation of diffuse astrocytic tumors [12]. We believe the major causes for the abovementioned inconsistency are the following: (1) there are huge physiological, pathological, and biological differences among various cancer types, specific pathologic processes, and so on which researchers focused on in their projects. (2) The selection of *b* values is significant for accurate fitting of the diffusion model, which further determines the calculation of parameters. (3) Data analysis can also contribute to the variance of conclusion. For instance, data analysis of hybrid IVIM-DKI is quite different from separate analysis of DKI and IVIM [22]. (4) Other potential factors include the number and distribution of patients.

There were several limitations in the present study. First, the sample size of patients was not large enough, and the number of patients was unbalanced in different histological grades. Particularly, the number of patients with WHO G1 rectal cancer is not enough. Second, the diagnosis was only applied, referring to the WHO criteria in the present study. However, other grading standards such as the poorly differentiated clusters called PDCs-based grading were not referred to further evaluate the diagnostic performance. Third, merely, the logistic regression was introduced to explore the integration of different DWI inspirations. Other complex statistical models should be introduced in the following work. Fourth, too large tumor may make it difficult to determine the tumor boundary and ultimately affect the accurate calculation of DWI-derived parameters.

In conclusion, this research indicates that greater diagnostic performance for grading rectal cancer can be obtained through integrating the DWI-derived cellularity, vascularity, and microstructural complexity quantified by parameters that were sourced from DKI and IVIM. Furthermore, because there were tight correlations among all DWI-derived biological inspirations, the integration of different biological inspirations of DWI holds great potential in achieving a more comprehensive tumor characterization, which will be meaningful for many other clinical applications including cancer treatment evaluation, tumor detection, and tumor recurrence prediction.

## Figures and Tables

**Table 1:** Patients characteristics.

Characteristics	Values
Mean age (years)	59.3 ± 10.2, (min: 40, max: 79)
Gender	
Male	33
Female	25
Histopathological grade	1–4
WHO-G1	11
WHO-G2	29
WHO-G3	18
Low grade (G1 and G2)	40
High grade (G3)	18
T stage
T1	2
T2	4
T3	34
T4	18
N stage	
N0	12
N1	28
N2	18
M stage	
M0	53
M1	5
Anatomic distribution	
Upper	17
Middle	28
Lower	13

**Table 2:** Diffusion parameters among different histologic grades.

	D (×10^−9^ m^2^/s)	*f*	*D* ^*∗*^ (×10^−9^ m^2^/s)	*D*_app_ (×10^−9^ m^2^/s)	*K*_app_
All subjects (*n* = 58)	1.282 ± 0.164	0.237 ± 0.070	45.458 ± 11.801	1.440 ± 0.201	0.739 ± 0.127
WHO grade 1 (*n* = 11)	1.465 ± 0.081	0.292 ± 0.067	40.329 ± 7.399	1.699 ± 0.099	0.604 ± 0.058
WHO grade 2 (*n* = 29)	1.323 ± 0.105	0.244 ± 0.052	45.622 ± 11.187	1.460 ± 0.127	0.715 ± 0.091
WHO grade 3 (*n* = 18)	1.105 ± 0.103	0.192 ± 0.072	48.329 ± 14.239	1.250 ± 0.144	0.862 ± 0.099
Low grade (1 and 2) (*n* = 40)	1.362 ± 0.117	0.257 ± 0.059	44.166 ± 10.470	1.526 ± 0.161	0.684 ± 0.096
High grade (3) (*n* = 18)	1.105 ± 0.103	0.192 ± 0.072	48.329 ± 14.239	1.250 ± 0.144	0.862 ± 0.099
*p* value (grade 1 vs. grade 2)	0.001	**0.079**	**0.412**	<0.001	0.003
*p* value (grade 1 vs. grade 3)	<0.001	<0.001	**0.182**	<0.001	<0.001
*p* value (grade 2 vs. grade 3)	<0.001	0.018	**0.721**	<0.001	<0.001
*p* value (low grade vs. high grade)	<0.001	0.002	**0.276**	<0.001	<0.001

^※^ *D*: true diffusion coefficient, *D*^*∗*^: pseudodiffusion coefficient, *D*_app_: apparent diffusion coefficient derived from DKI, *f*: perfusion fraction, *K*_app_: apparent diffusion kurtosis coefficient.

**Table 3:** ROC analysis results of different DWI inspirations and their combinations.

	Sensitivity (%)	Specificity (%)	AUC	Youden Index
*D*	94.4	87.5	0.912	0.819
*f*	77.8	92.5	0.843	0.703
*D*_app_	88.9	85.0	0.901	0.739
*K*_app_	88.9	85.0	0.910	0.739
*D* and *f*	94.4	87.5	0.946	0.819
*D* and *K*_app_	94.4	87.5	0.951	0.819
*f* and *K*_app_	88.9	87.5	0.901	0.764
*D* and *f* and *K*_app_	94.4	87.5	0.953	0.819

^※^ *D*: true diffusion coefficient, *D*^*∗*^: pseudodiffusion coefficient, *D*_app_: apparent diffusion coefficient derived from DKI, *f*: perfusion fraction, *K*_app_: apparent diffusion kurtosis coefficient.

## Data Availability

The data used to support the findings of this study are incorporated within the article.
